# A Non-obese, Treatment-Naive Japanese Diabetic Patient With Elevated Insulin Clearance and Hyperglycemia Under Enhanced Insulin Sensitivity and Increased Insulin Secretion: Elevated Insulin Clearance as Type 2 Japanese Diabetes Mellitus (T2JDM)

**DOI:** 10.7759/cureus.14354

**Published:** 2021-04-07

**Authors:** Seigo Sugiyama, Hideaki Jinnouchi, Kunio Hieshima, Noboru Kurinami, Katsunori Jinnouchi

**Affiliations:** 1 Diabetes Care Center, Jinnouchi Hospital, Kumamoto, JPN

**Keywords:** diabetes mellitus, insulin clearance, type 2 diabetes, insulin degrading enzyme, nonobese, hyperinsulinemic euglycemic clamp examination, insulin sensitivity, insulin resistance, hyperglycemia, c peptide immunoreactivity

## Abstract

Diabetes mellitus is a heterogeneous and complex metabolic disorder characterized by hyperglycemia secondary to either resistance to insulin actions on the liver and peripheral tissues, insufficient insulin secretion from pancreatic β-cells, or both. An integrated balance between blood insulin levels and whole-body insulin sensitivity could theoretically provide the clinical effectiveness of insulin action. Peripheral blood insulin concentrations might be determined by the capacity of endogenous pancreatic β-cell insulin secretion and the degree of the whole body insulin clearance. Here, we report a non-obese normoinsulinemic Japanese diabetic patient with elevated insulin clearance assessed by a hyperinsulinemic-euglycemic clamp examination and increased insulin secretion measured by daily urinary excretion of C-peptide immunoreactivity. We propose this unique pathogenic condition of diabetes with normoinsulinemia and elevated insulin clearance as “type 2 Japanese diabetes mellitus (T2JDM).”

## Introduction

Diabetes mellitus (DM) is clinically developed by mainly the pathogenic cause of impaired insulin actions. DM is known as a heterogeneous and complex metabolic disorder characterized by hyperglycemia secondary to resistance to insulin actions on the liver and peripheral tissues, insufficient endogenous insulin secretion from pancreatic β-cells, or both [1]. In patients with type 1 DM, severer pancreatic β-cell dysfunction rapidly leading to lowered blood insulin concentrations is the primary pathogenic cause of hyperglycemia. Because new-onset patients with type 2 DM (T2DM) usually have preserved capacity of endogenous insulin secretion from pancreatic β-cells, an integrated balance between blood insulin levels and whole-body insulin sensitivity could provide the clinical effectiveness of insulin, and then the totally decreased insulin actions can cause hyperglycemia.

Overweight and obesity are deeply associated with the development of T2DM but Asian and Japanese populations have a higher prevalence of T2DM than Caucasian and American subjects for the same body mass index (BMI) [2-3]. The mean BMI value in Asian T2DM patients is 23 kg/m^2^ [4], and we clinically found a number of non-obese T2DM patients in Japan. Japanese people are more likely to develop diabetes even if they are not obese. The development of T2DM with more significant pancreatic β-cell dysfunction evaluated by the blood concentration of insulin compared to Caucasians is considered as the responsible factor for the increased prevalence of T2DM in non-obese Japanese patients [5]. However, the clinical and pathogenic characteristics of non-obese Japanese T2DM patients have not been fully understood [6].

Here, we report the non-obese normoinsulinemic Japanese T2DM patient with elevated insulin clearance, increased insulin secretion, and enhanced insulin sensitivity.

## Case presentation

In December 2020, a 51-year-old non-obese Japanese man presented to the Diabetes Care Center at Jinnouchi Hospital in Kumamoto, Japan, because of the suspicion of T2DM with hyperglycemia by an annual health screening (fasting glucose; 164 mg/dL, hemoglobin A1c (HbA1c); 8.8 %). He was initially pointed out his hyperglycemia at fasting condition by a health screening three years ago (fasting glucose; 140 mg/dL, HbA1c; 7.6 %) but he did not go to the hospital because he had no symptoms and did not recognize his own disease severity. He was an office worker and did not have a habit of excessive soft drink intake, and an unbalanced diet caused zinc deficiency. He was a current smoker (10 cigarettes/day) and had a daily drinking habit (30 g of alcohol/day). He did not have an active exercise habit. He did not have a family history of T2DM, cardiovascular disease, or any habitual medications.

At his first visit to our outpatient service, clinical examination showed a body height of 168.1 cm, bodyweight of 62.2 kg, body mass index (BMI) of 22.2 kg/m^2^ (non-obese), blood pressure of 150/79 mmHg (hypertension), and regular pulse rate of 77 beats/min. Physical examination revealed no abnormalities. Laboratory examination showed hyperglycemia (fasting blood glucose concentration, 172 mg/dL); elevated concentration of HbA1c (8.5 %) with normoinsulinemia (4.2 μU/mL; normal rage: 2.2~12.4 μU/mL), high total cholesterol (248 mg/dL), and high triglyceride (342 mg/dL); and a reduced high-density lipoprotein cholesterol level (39 mg/dL) (Table 1).

**Table 1:** Laboratory data at the initial visit to Jinnouchi Hospital AST: aspartate aminotransferase, ALT: alanine aminotransferase, γGTP: γ glutamyl transpeptidase, LDH: lactate dehydrogenase, ALP: alkaline phosphatase, CPK: creatine phosphokinase, BUN: blood urea nitrogen, HDL: high-density lipoprotein, LDL: low-density lipoprotein, MCV: mean corpuscular volume, MCH: mean corpuscular hemoglobin, MCHC: mean corpuscular hemoglobin concentration

Biochemistry		Glucose metabolism	
Total protein (g/dL)	6.8	Fasting blood glucose (mg/dL)	172
Albumin (g/dL)	4.0	Hemoglobin A1c (%)	8.5
Total bilirubin (mg/dL)	0.5	Fasting Insulin (mU/mL)	4.2
AST (IU/L)	15		
ALT (IU/L)	17	[Blood cell count]	
g-GTP (IU/L)	33	White blood cells (/mL)	7690
LDH (IU/L)	153	Red blood cells (/mL)	504 x 10^4^
ALP (IU/L)	287	Hemoglobin (g/dL)	16.5
CPK (IU/L)	172	Hematocrit (%)	46.5
Amylase (IU/L)	63	MCV (fL)	92.3
Cholinesterase (IU/L)	320	MCH (pg)	32.7
Total-cholesterol (mg/dL)	248	MCHC (%)	35.5
HDL-cholesterol (mg/dL)	39	Platelets (/mL)	19.7 x 10^4^
Triglyceride (mg/dL)	342		
LDL-cholesterol (mg/dL)	137	[Urinary data]	
BUN (mg/dL)	11.6	pH	6.0
Creatinine (mg/dL)	0.84	Specific gravity	1.025
Uric acid (mg/dL)	6.4	Protein	-
Sodium (Na; mEq/L)	136	Glucose	+
Potassium (K; mEq/L)	3.7	Occult blood	-
Chloride (Cl; mEq/L)	101	Ketone	-
Calcium (Ca; mg/dL)	8.9	Urobilinogen	+/-
Zinc (Zn; μg/L)	780		

He did not have diabetic retinopathy, neuropathy, nephropathy, thyroid dysfunction, or adrenal abnormality. He was non-obese but not salcopenic, and he had no evidence of excess fat accumulation assessed by bioelectrical impedance analyzer [7] and abdominal computed tomography (Table 2).

**Table 2:** Anthropometric parameters of a non-obese patient before hospitalized treatments CT: computed tomography Elementary body composition was measured using a direct segmental multi-frequency bioelectrical impedance analyzer (InBody770; Biospace, Seoul, Korea).

		Assessment
Body Weight (kg)	62.2	-
Height (cm)	168.1	-
Body Mass Index (kg/m^2^)	22.2	Non-obese
Body Surface Area (m^2^)	1.67	Normal
Waist Circumstance (cm)	80.5	Normal
Body Fat Mass (kg)	13.7	Normal
Body Fat Percentage (%)	21.5	Normal
Skeletal Muscle Mass (kg)	28.1	Normal
Skeletal Muscle Mass Index (kg/m^2^)	7.5	Normal
Total Body Water (L)	36.6	Normal
Abdominal Visceral Fat Area by CT (cm^2^)	85.16	Normal
Abdominal Subcutaneous Fat Area by CT (cm^2^)	68.64	Normal
Total Abdominal Fat Area by CT (cm^2^)	153.80	Normal

We performed 75-gram oral glucose tolerance test (75g-OGTT) and we found the well-maintained basal insulin secretion without hyperinsulinemia, prolonged hyperglycemia, and poor elevating insulin response to glucose load (Table 3).

**Table 3:** Glucose and insulin concentrations in 75-g oral glucose tolerance test at the initial visit to Jinnouchi Hospital

	Pre-test	30 min	60 min	120 min
Glucose (mg/dL)	172	302	390	348
Insulin (µU/mL)	4.2	6.2	15.7	11.8

Based on the results of blood insulin levels in 75g-OGTT, we possibly speculated the presence of pancreatic β-cell dysfunction as his primary pathogenic cause of diabetes (Table 3).

Three weeks after his first visit, he was admitted to our hospital for assessment and treatment of his hyperglycemia. We found that his antibody titer to glutamic acid decarboxylase was undetectable (<5.0 U/mL) and his daily urinary C-peptide immunoreactivity (CPR) excretion was unexpectedly accelerated at 182.07 µg/day, thus establishing a definitive diagnosis of T2DM (Table 4).

**Table 4:** Glucose metabolic indexes from 75g-OGTT before hospitalized treatments HOMA-IR: homeostasis model assessment for insulin resistance, HOMA-beta: homeostatic model assessment for beta-cell function, QUICKI: quantitative insulin sensitivity check index, OGTT: oral glucose tolerance test, CPR: C-peptide immunoreactivity, 75g-OGTT: 75-gram oral glucose tolerance test

		Assessment
HOMA-IR	1.78	Normal
HOMA-beta	13.9	Decreased
Insulinogenic Index in 75g-OGTT	0.015	Decreased
Matsuda & DeFronzo Index in 75g-OGTT	6.19	Increased
QUICKI	0.35	Normal
CPR Index	1.61	Normal

After admission, we provided the hospitalized dietary (1800 Kcal/day) and exercise therapies according to our standard protocol [8]. On the second day of hospitalization (before treatment), his blood glucose levels were still elevated under the optimal dietary therapy (Table 5).

**Table 5:** Daily blood glucose profile, fasting insulin, and urinary CPR excretion before and after the hospitalized treatments CPR: C-peptide immunoreactivity

	Glucose Before Breakfast (mg/dL)	Glucose Post 2-Hr Breakfast (mg/dL)	Glucose Before Lunch (mg/dL)	Glucose Post 2-Hr Lunch (mg/dL)	Glucose Before Supper (mg/dL)	Glucose Post 2-Hr Supper (mg/dL)	Fasting Insulin (µU/mL)	Urinary CPR Excretion (µg/day)
On Admission	168	269	218	185	138	214	2.7	182.07
Post Hospitalized Treatment	107	125	96	127	105	113	1.8	103.75

On the third day of hospitalization, a hyperinsulinemic-euglycemic clamp examination [9] revealed no insulin resistance (M-value>8.0), enhanced insulin sensitivity (M/I>12.0), and elevated insulin clearance assessed by the metabolic clearance rate of insulin (MCRI>500) (Table 6).

**Table 6:** Assessment of diabetic condition and diabetes-associated complications at the pre-treatment state in hospitalization CPR: C-peptide immunoreactivity, GAD: glutamic acid decarboxylase, M-value: glucose infusion rate, M/I: (M-value)/(steady-state insulin), MCRI: metabolic clearance rate of insulin, eGFR: estimated glomerular filtration rate, CT: computed tomography, baPWV: brachial-ankle pulse wave velocity

	Measured Values / Finding	Assessment
Family History of Diabetes	No	Absence
Maximum Body Weight at 38 years old (kg)	65.0	-
Maximum Body Mass Index (kg/m^2^)	23.0	Normal
Fasting CPR (ng/mL)	2.43	Elevated
Fasting Insulin (μU/mL)	2.7	Normal
Molar Ratio of CPR/Insulin	42.9	Increased
Urinary CPR Excretion (μg/day)	182.1	Increased
Anti-GAD antibody (U/mL)	< 5.0	Negative
Euglycemic Hyperinsulimenic Clamp		
M-value (mg/kg/minute)	8.20	Normal
M/I (g•L/U/kg/minute)	13.06	Increased
Steady State Insulin (μU/mL)	62.8	Decreased
MCRI (mL/min/m^2^)	725.5	Increased
Retinopathy	None	Normal
Urinary Albumin Excretion (mg/day)	2.8	Normal
eGFR (mL/min/1.73m^2^)	75.9	Stage-1
Achilles Tendon Reflex	Right: +, Left: +	Normal
Liver / Spleen Attenuation Ratio in CT	1.15	Normal
Ankle Brachial Index	Right; 1.15, Left; 1.18	Normal
baPWV (cm/sec)	Right; 1280, Left; 1256	Normal

We also found the strongly increased hepatic insulin clearance assessed by the molar ratio of CPR/insulin at fasting condition (Table 6). Abdominal computed tomography showed normal subcutaneous and abdominal fat deposition and normal hepatic fat accumulation as assessed by the liver-to-spleen attenuation ratio (Table 6). His pathogenic condition of abnormal glucose-metabolism suggested the possibility of attenuated insulin actions by the elevated insulin clearance but neither pancreatic β-cell dysfunction nor insulin resistance.

Because of the preserved capacity of endogenous insulin secretion from pancreatic β-cells and the absence of insulin resistance, the patient initially received lifestyle modification therapy with stopping smoking and drinking, taking adequate calorie intake, reducing salt intake, and introducing an exercise habit during the hospitalized treatments [8]. He also received an explanation for his diabetic conditions and his novel pathogenesis of hyperglycemia with elevated insulin clearance. Because we have no established and evidence-based interventional strategies to treat his elevated insulin clearance, we started to treat him with metformin (initial dose of 500 mg/day and maintenance dose of 1000 mg/day) and followed up his hyperglycemia. His hyperglycemia was not life-threatening at the time of hospital admission (no ketosis and no symptom), and dietary therapy and metformin were effective for his hyperglycemia. During the hospitalized therapy, we measured fasting blood glucose levels every morning and measured the daily blood glucose profile. At the same time, we started statin (rosuvastatin; 5 mg/day), fibrate (fenofibrate; 160 mg/day), and angiotensin-converting enzyme inhibitor (enalapril; 5 mg/day) to treat his hyperlipidemia and hypertension, and we subsequently added ezetimibe 10 mg/day and α-glycosidase inhibitor (voglibose; 0.9 mg/day). On the fourteenth day of hospitalization, his blood glucose concentration and blood pressure (blood pressure; 119/71 mmHg) were well-controlled (Table 6). We finally found a decrease in daily urinary CPR excretion (103.75 µg/day) and blood insulin level (1.8 μU/mL) as compared to the pre-treatment condition. His elevated hepatic insulin clearance was not rapidly changed after the two weeks of hospital therapies (molar ratio of CPR/insulin at fasting condition; 41.8). After 17 days of in-hospital therapies and education, the patient was discharged with the recommendation to continue appropriate dietary therapy (1800 kcal/day as described above) and at least 30 minutes of daily exercise. The patient continued treatment with all the medications described above and underwent monthly outpatient follow-ups.

After discharge, we followed him at our outpatient service as usual [8], which led to an improvement in his blood glucose and glycated hemoglobin (HbA1c) concentrations. In March 2021, his blood glucose, blood pressure, and lipid parameters have been well managed at our outpatient service (fasting glucose; 117 mg/dL, and HbA1c; 6.6 %, blood pressure; 112/71 mmHg, low-density lipoprotein cholesterol; 59 mg/dL, and triglyceride; 75 mg/dL). We want to re-perform the hyperinsulinemic-euglycemic clamp examination to evaluate insulin clearance and insulin sensitivity at our outpatient service after 12 months of treatment or after glycemic control had been improved. We are not sure whether we will be able to re-evaluate the elevated insulin clearance in the future.

## Discussion

In the present case, we initially found a treatment-naive, non-obese, normoinsulinemic Japanese patient with hyperglycemia and paradoxically increased endogenous insulin secretion. Hyperinsulinemic-euglycemic clamp examination revealed the increased insulin sensitivity. The patient exhibited greatly elevated hepatic insulin clearance assessed by the fasting molar ratio of CPR/insulin and whole-body insulin clearance assessed by MCRI in the clamp examination.

DM is developed by the mainly pathogenic cause of impaired insulin actions. DM has been known as a heterogeneous and complex metabolic disorder characterized by hyperglycemia secondary to either resistance to the insulin actions on the liver and peripheral tissues, insufficient insulin secretion from pancreatic β-cells, or both [1]. Because new-onset patients with T2DM usually have preserved capacity of endogenous insulin secretion from pancreatic β-cells, an integrated balance between blood insulin levels and whole-body insulin sensitivity could provide the clinical effectiveness of insulin, and then the totally decreased insulin actions can cause hyperglycemia [1]. Regarding the efficacy of insulin actions, blood insulin levels play crucial roles in glucose metabolism, and insulin clearance is a very important pathogenic factor to determine blood glucose levels in the early phase of T2DM [10]. Our present non-obese case exhibited normoinsulinemic hyperglycemia under increased endogenous insulin secretion, suggesting that his elevated insulin clearance could be importantly involved in this pathogenesis. To the best of our knowledge, there are no cases reporting elevated insulin clearance in a treatment-naive, non-obese patient with T2DM. We speculate that the elevated insulin clearance could be the primary cause of this pathological condition but we have no evidence now. We have no established and appropriate treatment/management strategy to modify the elevated insulin clearance. We will retrospectively investigate the clinical characteristics of treatment-naive T2DM patients with elevated insulin clearance in the next study. We hope that we can successfully report the results in the near future.

Hepatic insulin clearance, a physiological process that in response to nutritional cues clears more than half of circulating insulin secreted from pancreatic β-cells into the portal vein, is emerging as an important factor in our understanding of the pathogenesis of T2DM [10]. In extra-hepatic insulin clearance, the kidney, muscle, and other organs can degrade insulin [11]. Insulin-degrading enzyme (IDE) is a ubiquitously expressed and highly conserved Zn^2+^-metalloprotease that degrades insulin and several other intermediate-size peptides in the liver and peripheral tissues and can primarily involve in the whole body insulin clearance [12]. Insulin clearance is a highly heritable trait [13], raising the possibility that genetic determinants of insulin clearance may affect the risk for developing DM in Japanese [14]. It has been previously suggested that controlling IDE activity could provide yet another potential therapeutic approach in diabetes [15-16]. We have no information regarding the possible involvement of augmented IDE activity in his elevated insulin clearance because we could not measure the blood concentrations of IDE and the enzymatic activity of IDE in the present case. Future works will need to delineate the molecular pathways and mechanisms mediating the changes in insulin clearance in non-obese patients with T2DM.

It has been reported that patients with hyperthyroid and newly diagnosed diabetes exhibited a marked increase in insulin clearance and the increased insulin clearance became normal with the amelioration of the hyperthyroid state [17]. In our case, the patient had no abnormality in the thyroid function. Previously, Vasquez et al. reported elevated insulin clearance in obese Pima-Indians with non-insulin-dependent diabetes [18] but their patients were extremely obese (mean BMI; 42.2 kg/m^2^) and had elevated fasting insulin concentrations and dysfunction of pancreatic β-cells. Then, the pathogenic condition between the Pima-Indians and our case should be different. At the present time, we do not know the potential involvement of the elevated insulin clearance in the pathogenesis of the abnormal glucose metabolism in Japanese patients with T2DM. Further studies are needed to investigate the frequency of T2DM patients, especially non-obese T2DM patients, with elevated insulin clearance in real clinical practice.

Salsalate is a non-steroidal anti-inflammatory drug shown to improve glucose tolerance in DM patients [19], and salsalate treatment has been shown to decrease insulin clearance and increase the plasma insulin concentration associated with a decrease in fasting glucose concentration in insulin-resistant individuals without diabetes [20]. The exact mechanism by which salsalate could decrease insulin clearance is unknown, but it raises the possibility that drugs might be identified that would be helpful in the treatment of T2DM with elevated insulin clearance by directly modifying insulin clearance. Unfortunately, we did not actually have any chances to treat this patient with salsalate because salsalate is prohibited from being prescribed in Japanese clinical practice.

This case was not one of zinc deficiency (Table 1). We did not analyze the gene mutation of zinc transporter 8 (ZnT8). Because our case had a well-preserved capacity of insulin secretion from pancreatic β-cells and had no family history of diabetes, we speculate this case could not have mutations in ZnT8.

Because we had no established and evidence-based interventional strategies to treat his elevated insulin clearance, we started to treat him with metformin and α-glycosidase inhibitor, both of which are capable to lower blood glucose concentration without augmentation of insulin secretion. The ongoing clinical course of treating his diabetes is doing well in our outpatient service, however, we need to watch and monitor his diabetic condition carefully, and we will also want to re-examine the changes in the state of his elevated insulin clearance in the future.

## Conclusions

This diabetic case potentially suggested a novel and crucial pathogenesis of the onset of T2DM caused by elevated insulin clearance resulting in decreased peripheral blood insulin concentrations and leading to impaired insulin actions. We propose this unique pathogenic condition in non-obese diabetes with normoinsulinemia and elevated insulin clearance as “type 2 Japanese diabetes mellitus (T2JDM).”
